# Iminosugar Amino Acid idoBR1 Reduces Inflammatory Responses in Microglia

**DOI:** 10.3390/molecules27103342

**Published:** 2022-05-23

**Authors:** Olumayokun A. Olajide, Victoria U. Iwuanyanwu, Owolabi W. Banjo, Atsushi Kato, Yana B. Penkova, George W. J. Fleet, Robert J. Nash

**Affiliations:** 1 Department of Pharmacy, School of Applied Sciences, University of Huddersfield, Queensgate, Huddersfield HD1 3DH, UK; o.a.olajide@hud.ac.uk (O.A.O.); victoria.iwuanyanwu@hud.ac.uk (V.U.I.); owolabi.banjo@hud.ac.uk (O.W.B.); 2 Department of Hospital Pharmacy, University of Toyama, 2630 Sugitani, Toyama 930-0194, Japan; kato@med.u-toyama.ac.jp; 3 Phytoquest Limited, Plas Gogerddan, Aberystwyth SY23 3EB, UK; y.penkova@phytoquest.co.uk; 4 Chemistry Research Laboratory, Department of Chemistry, University of Oxford, Oxford OX1 3TA, UK; george.fleet@sjc.ox.ac.uk

**Keywords:** (2*R*,3*R*,4*R*,5*S*)-3,4,5-trihydroxypiperidine-2-carboxylic acid, iminosugar amino acid-idoBR1, *Cucumis sativus*, microglia cell line, lipopolysaccharide, pro-inflammatory factors, neuroprotection

## Abstract

(1) Background. Inflammation is reported to be a key factor in neurodegeneration. The microglia are immune cells present in the central nervous system; their activation results in the release of inflammatory cytokines and is thought to be related to aging and neurodegenerative disorders, such as Alzheimer’s disease. (2) Methods. A mouse BV-2 microglia cell line was activated using LPS and the anti-inflammatory cucumber-derived iminosugar amino acid idoBR1, (2*R*,3*R*,4*R*,5*S*)-3,4,5-trihydroxypiperidine-2-carboxylic acid, was used alongside dexamethasone as the control to determine whether it could reduce the inflammatory responses. (3) Results. A dose-dependent reduction in the LPS-induced production of the proinflammatory factors TNFα, IL-6, and nitric oxide and the transcription factor NF-*κ*B was found. (4) Conclusions. Further investigations of the anti-inflammatory effects of idoBR1 in other models of neurodegenerative diseases are warranted.

## 1. Introduction

Inflammation is reported to be a key factor in neurodegeneration [1,2]. Cucumbers and cucumber extracts have long been recognized as having anti-inflammatory properties, and have been used topically for various types of skin problem, including swelling under the eyes and sunburn [3]. There are many anecdotal claims on the internet that cucumbers (*Cucumis sativus*) are beneficial to brain function. These claims are largely supported by the presence of the anti-inflammatory flavonoid fisetin. However, the calculated daily intake of dietary fisetin from fruit (0.4 mg/day) is much lower than the daily recommendation (100 mg/day) [4]. While fisetin may not be the responsible compound in cucumbers, cucumbers do contain an anti-inflammatory iminosugar amino acid, idoBR1, (2*R*,3*R*,4*R*,5*S*)-3,4,5-trihydroxypiperidine-2-carboxylic acid (**1**), which is orally available and stable in vivo [5]. The iminosugar idoBR1 was shown to reduce LPS-induced pro-inflammatory cytokine tumour necrosis factor alpha (TNFα) in both ex vivo human serum and the human monocyte-like THP-1 cell line [5]. TNFα can drive degenerative changes when chronically elevated. Various studies have revealed that the manipulation of TNFα, one of the few gliotransmitters, might have important effects on diseases characterized by glial activation, cytokine-mediated neuroinflammation, and synaptic dysfunction [6]. In this study, we investigated the ability of idoBR1 to reduce the release of proinflammatory factors by mouse microglia in culture; microglia are the only immune cells in the parenchyma of the central nervous system and in close proximity to neurons [7]. In neuroinflammation, microglia become activated, undergo a change in morphology, and release various cytotoxic mediators. Microglia are thought to be important factors in brain aging and pathologies, including Alzheimer’s and Parkinson’s [8,9]. They are known to release pro-inflammatory cytokines, such as TNF*α*, when activated, as well as potentially neurotoxic substances, such as nitric oxide.

**Scheme 1:**
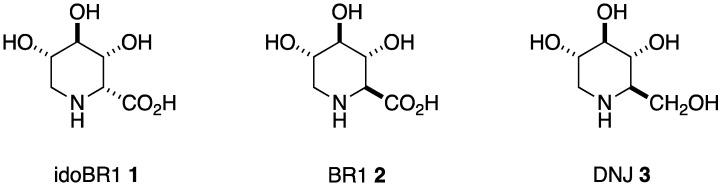
Piperidine iminosugar amino acids and their iminosugar analogue.

Of the very few iminosugar acids reported as natural products, BR1 (2*S*,3*R*,4*R*,5*S*)-3,4,5-trihydroxypiperidine-2-carboxylic acid) (**2**), from the African tree *Baphia racemosa*, was the first to be isolated [10]. Iminosugar acids are much more difficult to isolate and identify than their iminosugar analogues, e.g., DNJ (**3**), due to the high content of acidic and neutral amino acids in plants [11] (Scheme 1). idoBR1 (**1**), even though it was found in a common food plant, was synthesized before it was isolated [12]. BR1 is a β-glucuronidase inhibitor, but idoBR1 is not; neither inhibit glucosidases in the manner of the related iminosugar DNJ. Cucumber extract standardized to >1% idoBR1 has shown benefits in osteo-arthritis at low doses [13], without signs of toxicity [14]. DNJ has been reported to have neuroprotective effects in a mouse Parkinson’s model when used with Ibuprofen [15]. DNJ and derivatives as potent glucosidase inhibitors can produce side effects [16]. We were interested in testing whether idoBR1, without glucosidase inhibition, might also have the potential to reduce the release of inflammatory signals by microglia and, therefore, whether it would display neuroprotective activity. Both DNJ and idoBR1, as small sugar analogues, are likely to pass the blood–brain barrier [17].

## 2. Results

### 2.1. The Iminosugar idoBR1 Reduced Nitric Oxide Production in LPS-Stimulated BV-2 Microglia

**Figure 1:**
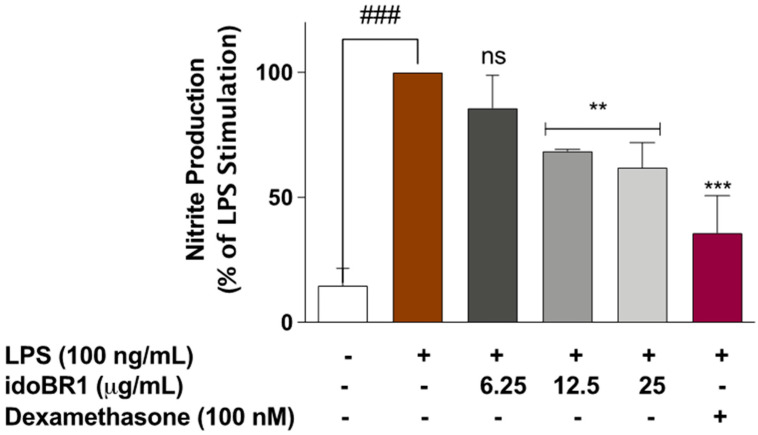
Pre-treatment with idoBR1 (12.5 and 25 μg/mL) and dexamethasone (100 nM) reduced nitrite production in BV-2 microglia stimulated with LPS (100 ng/mL) for 24 h. Nitrite production was determined in culture supernatants obtained from BV2 microglia using Griess assay. All values are expressed as mean ± SEM for three independent experiments. Data were analyzed using one-way ANOVA for multiple comparisons with post hoc Student Newman–Keuls test. ns (not significant); ** *p* < 0.01; *** *p* < 0.001 (treatment vs. LPS stimulation only); ^###^ *p* < 0.001 (unstimulated cells vs. stimulated with LPS only).

In experiments conducted to evaluate the effects of idoBR1 on nitric oxide (NO) release (nitrite production), stimulation of BV-2 microglia with LPS (100 ng/mL) resulted in a significant (*p* < 0.001) elevation (*p* < 0.001) in nitrite production, compared to unstimulated cells. In the presence of idoBR1 (6.25 µg/mL), reduction in nitrite production was not significant (*p* < 0.05). However, with 12.5 and 25 µg/mL of idoBR1, nitrite production was significantly reduced (*p* < 0.01), by 31% and 38%, respectively, compared to nitrite production with LPS alone. Pre-treatment with dexamethasone (100 nM) resulted in a ~64% reduction in nitrite production (Figure 1).

### 2.2. The Production of TNFα and IL-6 Was Reduced by idoBR1 Treatment

**Figure 2:**
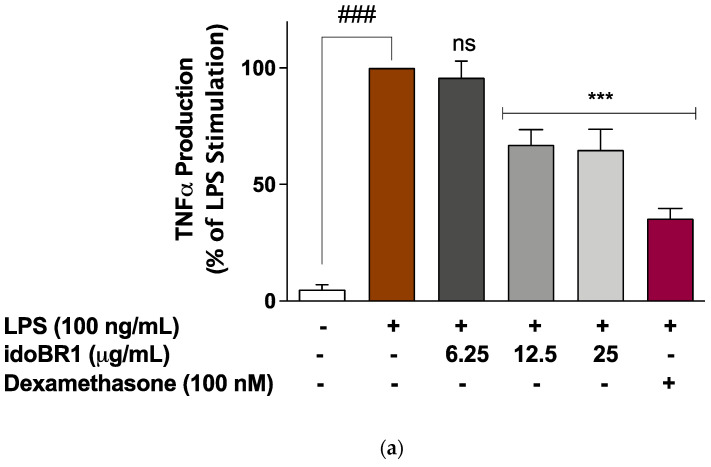

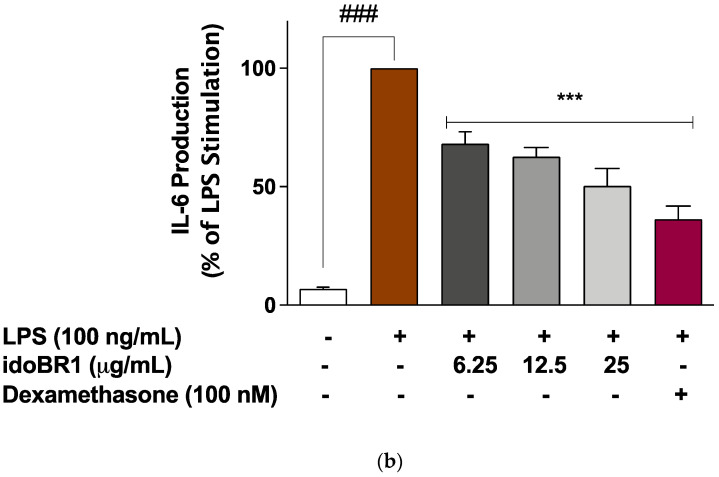
(**a**). Pre-treatment with idoBR1 (12.5 and 25 μg/mL) and dexamethasone (100 nM) reduced TNFα production in BV-2 microglia stimulated with LPS (100 ng/mL) for 24 h. TNFα production was evaluated in culture supernatants obtained from BV-2 microglia using mouse TNFα ELISA. All values are expressed as mean ± SEM for three independent experiments. Data were analyzed using one-way ANOVA for multiple comparisons with post hoc Student Newman–Keuls test. ns (not significant); *** *p* < 0.001 (treatment vs. LPS stimulation only); ^###^ *p* < 0.001 (unstimulated cells vs. cells stimulated with LPS only). (**b**). Pre-treatment with idoBR1 (6.25, 12.5, and 25 μg/mL) and dexamethasone (100 nM) reduced IL-6 production in BV-2 microglia stimulated with LPS (100 ng/mL) for 24 h. IL-6 production was evaluated in culture supernatants obtained from BV2 microglia using mouse IL-6 ELISA. All values are expressed as mean ± SEM for three independent experiments. Data were analyzed using one-way ANOVA for multiple comparisons with post hoc Student Newman–Keuls test. ns (not significant); *** *p* < 0.001 (treatment vs. LPS stimulation only); ^###^ *p* < 0.001 (unstimulated cells vs. cells stimulated with LPS only).

The increased release of TNFα following the stimulation of the BV-2 microglia with LPS (100 ng/mL) was significantly (*p* < 0.001) reduced in the presence of idoBR1 (12.5 and 25 µg/mL) and dexamethasone (100 nM), while the pre-treatment with the lowest concentration of idoBR1 (6.25 µg/mL) did not prevent TNFα production (Figure 2a). The elevation in IL-6 production observed after the LPS stimulation of the BV-2 microglia was significantly reduced in a concentration-dependent fashion by the idoBR1 (6.25, 12.5, and 25 µg/mL). A reduction in IL-6 production was observed in the cells pre-treated with dexamethasone (100 nM) prior to stimulation with LPS (Figure 2b).

### 2.3. The Iminosugar idoBR1 Attenuated the Activation of NF-κB in LPS-Stimulated BV-2 Microglia

**Figure 3:**
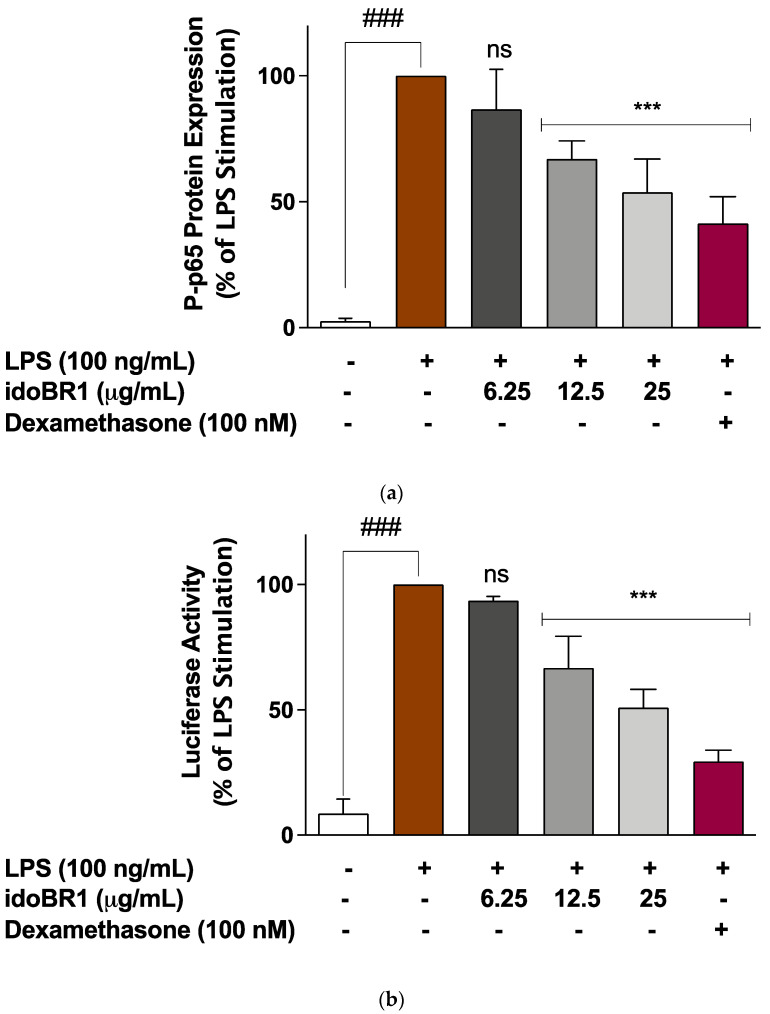

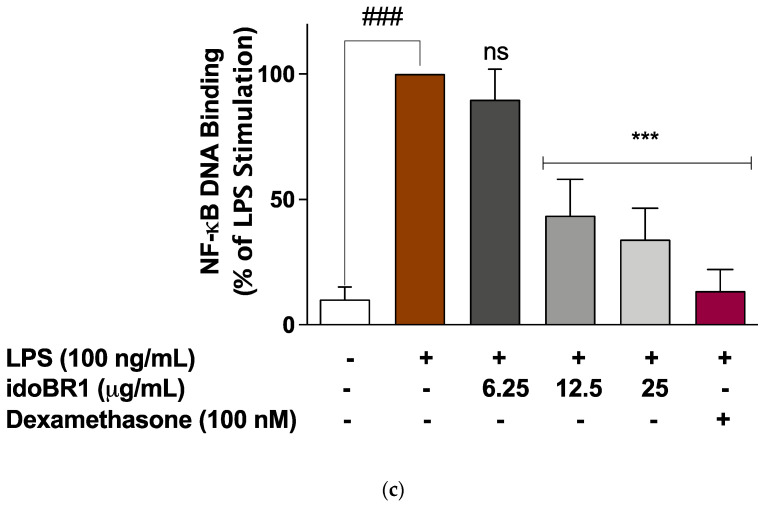
(**a**). idoBR1 (12.5 and 25 μg/mL) and dexamethasone (100 nM) suppress neuroinflammation by reducing LPS-induced increased phosphorylation of NF-*κ*B p65 sub-unit in BV-2 microglia. Protein levels of phospho-NF-*κ*B p65 (Ser536) sub-unit were determined in lysates obtained from BV-2 microglia stimulated with LPS for 60 min using ELISA. Values are expressed as mean ± SEM for three independent experiments. Data were analyzed using one-way ANOVA for multiple comparisons with post hoc Student Newman–Keuls test. ns (not significant); *** *p* < 0.001 (treatment vs. LPS stimulation only); ^###^ *p* < 0.001 (unstimulated cells vs. cells stimulated with LPS only). (**b**). idoBR1 (12.5 and 25 μg/mL) and dexamethasone (100 nM) interfered with NF-*κ*B activity through inhibition of NF-κB luciferase activity in BV-2 cells transfected with pGL4.32(luc2P/ NF-*κ*B-RE/Hygro) vector and stimulated with LPS (100 ng/mL) for 6 h. Values are expressed as mean ± SEM for three independent experiments. Data were analyzed using one-way ANOVA for multiple comparisons with post-hoc Student Newman–Keuls test. ns (not significant); *** *p* < 0.001 (treatment vs. LPS stimulation only); ^###^ *p* < 0.001 (unstimulated cells vs. cells stimulated with LPS only). (**c**) LPS-induced increased DNA binding by NF-*κ*B was reduced by idoBR1 (12.5 and 25 μg/mL) and dexamethasone (100 nM). Nuclear extracts from BV-2 cells stimulated with LPS (100 ng/mL) for 60 min were investigated for binding to NF-*κ*B consensus site (5′-GGGACTTTCC-3′). Values are expressed as mean ± SEM for three independent experiments. Data were analyzed using one-way ANOVA for multiple comparisons with post hoc Student Newman–Keuls test. ns (not significant); *** *p* < 0.001 (treatment vs. LPS stimulation only); ^###^ *p* < 0.001 (unstimulated cells vs. cells stimulated with LPS only).

The results of the ELISA experiments used to evaluate the effects of idoBR1 on the cytoplasmic activation of NF-*κ*B revealed that following the stimulation of the BV-2 with the LPS microglia for 60 min, there was a significant (*p* < 0.001) increase in the protein levels of the phosphor-NF-*κ*B p65 (Ser536) sub-unit. Pre-treatment of the cells with idoBR1 (6.25 µg/mL) did not result in a significant reduction in elevated phosphor-NF-*κ*B p65 protein. However, in the presence of 12.5 and 25 µg/mL of idoBR1, as well as dexamethasone (100 nM), the increased protein expression of phosphor-NF-*κ*B p65 was significantly (*p* < 0.001) reduced (Figure 3a). Further experiments revealed that in the presence of 12.5 and 25 µg/mL of idoBR1, LPS-induced increased DNA binding of NF-*κ*B p65 to consensus sites, as well as the increased NF-*κ*B luciferase activity, were significantly reduced (Figure 3b,c).

### 2.4. The Effects of idoBR1 on the Viability of LPS-Stimulated BV-2 Microglia

**Figure 4:**
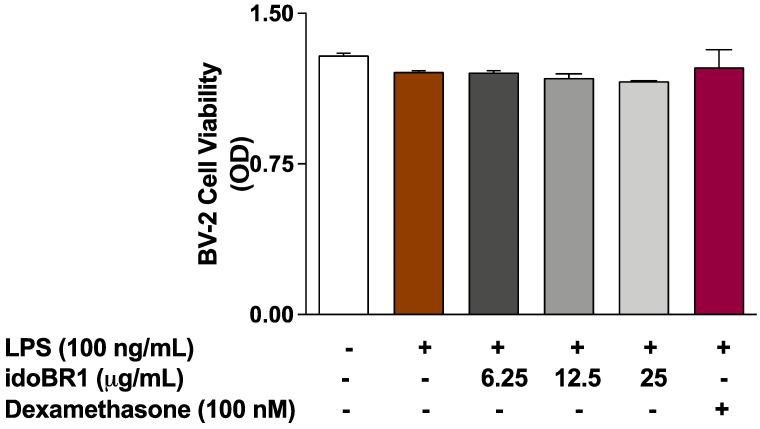
MTT assay showing effects of idoBR1 (6.25, 12.5, and 25 μg/mL) and dexamethasone (100 nM) on the viability of BV-2 microglia stimulated with LPS (100 ng/mL) for 24 h.

The results of the MTT assay in Figure 4 show that pre-treatment with 6.25, 12.5, and 25 µg/mL of idoBR1, followed by stimulation with LPS for 24 h, did not reduce the viability of the BV-2 microglia.

## 3. Discussion

The purpose of this study was to determine whether the anti-inflammatory iminosugar amino acid idoBR1 found in Cucumis sativus [5] could reduce the release of LPS-induced pro-inflammatory factors through a microglial cell line. Microglia are the resident immune cells of the brain. They are known to release pro-inflammatory cytokines, such as IL-6 and TNF*α*, when activated, as well as potentially neurotoxic substances, such as nitric oxide [1,2]. As previously reported with monocyte-like THP-1 cells and human serum, idoBR1 was shown to be able to reduce LPS-induced TNFα in BV-2 microglia cells [5]. In addition, we have shown here that idoBR1 reduced LPS-induced IL-6 and nitric oxide and decreased the activation of the transcription factor, nuclear factor kappa B (NF-*κ*B), without affecting the cells’ viability. The inflammation resulting from the activation of microglia appears to be involved in various neurodegenerative CNS disorders, including Alzheimer’s disease (AD), as well as PD [2]. Increased TNFα and IL-6 in the cerebrospinal fluid (CSF) are also reported to be associated with the pathology of depression in systemic lupus erythematosus [18].

Apart from the recent report on DNJ combined with Ibuprofen [15], iminosugars have not been widely reported to have anti-inflammatory activity, but the synthetic sp^2^-iminosugar glycolipid 1-dodecylsulfonyl-5*N*,6*O*-oxomethylidenenojirimycin (DSO 2-ONJ), has been reported to have potential in the treatment of diabetic retinopathy, in which neuroinflammation and microglia polarization are early events [19]. Synthetic glucosidase inhibitors (DNJ derivatives) have been reported to modulate Interferon gamma and TNFα receptor signalling in Dengue virus infection in primary human macrophages; this is possibly related to *N*-linked glycosylation changes [20]. In addition, it was proposed that these glucosidase-inhibiting iminosugars can control inflammation in viral, bacterial, and fungal sepsis [21]. The iminosugar idoBR1 is not a glucosidase inhibitor but, as a natural anti-inflammatory food iminosugar amino acid [5], it is of particular interest due to its possible dietary health benefits.

NF-*κ*B has been shown to control inflammatory responses in microglial cells. Therefore, it is notable that idoBR1 seems able to reduce the activation of NF-*κ*B. The activation of NF-*κ*B is triggered by phosphorylation and the subsequent proteasome-mediated degradation of the inactive complex I*κ*B. This process leads to the translocation of the free NF-*κ*B to the nucleus, where it promotes the expression of pro-inflammatory genes, such as the pro-inflammatory cytokines TNF*α* and IL-6, as well as inducible nitric oxide synthase (iNOS) [22]. NF-*κ*B has been shown to be expressed in the brains of AD patients, along with high levels of pro-inflammatory cytokines, which are thought to be major contributors to the pathogenesis of AD [23]. The inhibition of NF-*κ*B is also suggested as a target for reducing neuroinflammation in Parkinson’s disease [24].

The decrease in LPS-stimulated nitric oxide release is also notable because its excessive production is associated with both acute and chronic inflammation [25]. Neurons are highly susceptible to nitric-oxide-induced death, and very low concentrations can cause extensive neuronal damage and death. It seems likely, however, that idoBR1 could be effective at reducing the release of nitric oxide and other inflammatory factors through activated microglia by reducing the activation of NF-*κ*B. 

There is mounting evidence that microglia protect against the incidence of AD, as impaired microglial activities and altered microglial responses to β-amyloid are associated with increased AD risk; activated microglia also appear to be harmful to neurons and can exacerbate tau pathology and secrete inflammatory factors that can injure neurons, either directly or through the activation of neurotoxic astrocytes [23]. Anti-inflammatory approaches are recognized as having the potential to delay the onset or slow the progression of both AD and PD [23,24,26]. However, new approaches seem to be required, since the long-term use of steroids is not recommended and commonly used non-steroidal anti-inflammatory drugs (NSAIDs) can have negative effects in several areas of the body, including on the central nervous system [27,28]. The perispinal administration of anti-TNF antibodies, such as Etanercept, led to rapid improvements in Alzheimer’s patients [26], but the immune response to these proteins severely limits their prolonged use [29]. The treatment of AD and other neurodegenerative disorders with anti-inflammatory agents is challenging; this is partly due to the blood–brain barrier, which blocks the entry of molecules from the blood into the brain. Although further work is required to prove its ability to cross the blood–brain barrier, idoBR1 appears to be promising as a new anti-inflammatory agent that can decrease NF-*κ*B activation and, thereby, reduce the production of pro-inflammatory factors, such as TNFα.

Most of the anti-inflammatory drugs that have been investigated in clinical trials for AD are known to target specific single inflammatory mechanisms [1]. It is therefore highly interesting that DNJ in combination with Ibuprofen seemed to give positive results in the mouse Parkinson’s disease model. The results presented here suggest that iminosugars that are not glucosidase inhibitors (with probable off-target activities) may have potential in multi-target approaches to neurodegenerative disorders. 

The iminosugar idoBR1 was reported to be able to reduce the binding of hyaluronic acid to CD44 and to reduce the activity of an induced sialidase related to TNFα production [5]. It also seemed able to reduce the expression of the p38 mitogen-activated protein kinase (MAPK) in monocyte-like THP-1 cells. The p38 MAPK has been implicated in neuroinflammation [30]. Evidence linking p38 MAPK to neuroinflammation has been reported, suggesting that the exposure of microglia to Aβ induces microglial activation, subsequently leading to the production of neurotoxic pro-inflammatory cytokines and reactive oxygen species, which, in turn, activate p38 MAPK signaling [31]. Aβ-induced oxidative stress also results in the activation of p38 MAPK, with resultant tau hyperphosphorylation. Reports also suggest that p38 MAPK plays a role in neuroinflammation and AD due to its ability to activate NF-*κ*B [31], making it a potential molecular target for novel AD treatment. The sp^2^-iminosugar, DSO 2-ONJ, is reported to activate p38 (albeit while decreasing the activation of NF-κB), perhaps by interacting with the lipid-binding site of the protein [19]. As a highly polar molecule, idoBR1 is not likely to have the same binding properties. The iminosugar idoBR1 appears to have a novel anti-inflammatory mechanism involving sialidase and the reduction of both NF-*κ*B and p38, and it could be worthy of further investigation in models of neurodegenerative diseases generally. Related small iminosugar amino acids and iminosugars should also be studied further, as they appear to be readily orally available and stable in vivo, with low toxicity, and their similarity to common sugars may mean they are actively transported around the body [17]. A clinical trial on osteoarthritis using just 20 mg per day of cucumber extract standardized to >1% idoBR1 indicated that the molecule could be highly effective against inflammatory disorders [13]. 

## 4. Materials and Methods

The idoBR1 was purchased from Biosynth Carbosynth, Newbury, UK.

### 4.1. Cell Culture

BV-2 mouse microglia cell line ICLCATL03001 was obtained from Interlab Cell Line Collection (Banca Biologica e Cell Factory, Genova, Italy) and maintained in RPMI1640 medium supplemented with 10% fetal bovine serum (FBS) (Sigma-Aldrich, Gillingham, UK), 1 mM sodium pyruvate (Sigma), and penicillin/streptomycin (Sigma). When confluent, cells were split 1:10 using trypsin/EDTA solution and cultured at 37 °C in 5% CO_2_ incubator.

### 4.2. MTT Cell Viability Assay

BV-2 microglia were treated with idoBR1 (6.25, 12.5, and 25 µg/mL) for 30 min and then incubated with LPS (100 ng/mL) for 24 h. Subsequently, the cell culture medium was removed and replaced with MTT solution (5 mg/mL), followed by incubation at 37 °C for 4 h. Thereafter, 150 µL of MTT solution was removed from each well and replaced with 150 µL DMSO. Formazan crystals were dissolved by shaking the plate on a rocker. Absorbance was read at 570 nm using a Tecan Infinite Nano microplate reader.

### 4.3. Determination of Nitrite Production

BV-2 cells were pre-treated with idoBR1 (6.25, 12.5, and 25 µg/mL). Thirty minutes later, the cells were stimulated with LPS (100 ng/mL) for an additional 24 h. Production of nitrite in culture supernatants was determined using a Griess assay kit (Promega, Southampton, UK), according to the manufacturer’s instructions. Absorbance was read at 540 nm using a Tecan Infinite Nano microplate reader. The effects of idoBR1 were compared with those of dexamethasone (100 nM).

### 4.4. Determination of TNFα and IL-6 Production

Cultured BV-2 microglia were treated with idoBR1 (6.25, 12.5, and 25 µg/mL). After 30 min, cells were stimulated with LPS (100 ng/mL) for 24 h. Dexamethasone (100 nM) was used a reference drug. Levels of TNF*α* and IL-6 in culture supernatants were measured using mouse ELISA kits (Biolegend, San Diego, CA, USA), according to the manufacturer’s instructions. Absorbance was measured at a wavelength of 450 nm.

### 4.5. ELISA for Phospho-NF-κB p65 (Ser536) Sub-Unit

Cultured BV-2 microglia were treated with idoBR1 (6.25, 12.5, and 25 µg/mL). After 30 min, cells were stimulated with LPS (100 ng/mL) for 60 min. Dexamethasone (100 nM) was used a reference drug. Protein levels of phospho-NF-*κ*B p65 (Ser536) in cell lysates were measured using PathScan^®^ Phospho-NF-*κ*B p65 (Ser536) sandwich ELISA antibody pair (Cell Signaling Technology, Danvers, MA, USA), according to the manufacturer’s instructions. Absorbance was measured at a wavelength of 450 nm.

### 4.6. Transient Transfection and NF-κB Reporter Gene Assay

BV-2 cells were transfected with Cignal NF-*κ*B luciferase reporter using magnetofection (OZ Biosciences, Marseille, France) and incubated for 20 h at 37 °C in 5% CO_2_ incubator. At the end of the incubation period, cells were stimulated with LPS (100 ng/mL) in the presence or absence of idoBR1 (6.25, 12.5, and 25 µg/mL) for 6 h. Luciferase activity was determined with a Dual-Glo luciferase assay kit (Promega, Southampton, UK). Luminescence was measured using FLUOstar OPTIMA plate reader.

### 4.7. NF-κB Transcription Factor Binding Assay

DNA binding capacity of NF-*κ*B following activation by either LPS was investigated using an ELISA-based NF-*κ*B transcription factor kit (Abcam, Cambridge, UK) containing a 96-well plate, at which the NF-*κ*B consensus site (5′-GGGACTTTCC-3′) oligonucleotide was immobilized. Following treatment of BV-2 microglia with idoBR1 (6.25, 12.5, and 25 µg/mL) for 30 min, cells were stimulated with LPS (100 ng/mL) for another 60 min. Nuclear extracts from the cells were analyzed in DNA binding assays according to the manufacturer’s instructions. Absorbance was read at 450 nm.

### 4.8. Statistical Analysis

The values of all the results are represented as the mean ± SEM of at least three experiments. Data were analyzed using one-way analysis of variance followed by a post hoc Tukey test. Statistical differences of *p* values of less than 0.05 (*p* < 0.05) were considered significant.

## Data Availability

Not applicable.

## Sample Availability

Samples of idoBR1 are available from the authors.
